# Palau: Non-Communicable Disease Off-Island Treatment Referrals 2020–2022

**DOI:** 10.3390/ijerph22030431

**Published:** 2025-03-14

**Authors:** Quan Lac, Yujin Na, Kennedy Kainoa Tamashiro, Kelley Withy, Myra Adelbai-Fraser, Catherine Decherong, Greg Dever

**Affiliations:** 1 John A. Burns School of Medicine, University of Hawaiʻi, Honolulu, HI 96813, USA; yujinna@hawaii.edu (Y.N.); kkzt@hawaii.edu (K.K.T.); withy@hawaii.edu (K.W.); 2 Hawaii/Pacific Basin Area Health Education Center, Honolulu, HI 96813, USA; 3 Bureau of Hospital & Clinical Services, Koror 96940, Palau; madelbai@gmail.com; 4 Palau Area Health Education Center, Koror 96940, Palau; palchc@gmail.com (C.D.); gregd@pihoa.org (G.D.)

**Keywords:** Palau, non-communicable disease, medical referrals, economic impact, oncology, cardiology

## Abstract

**Background:** The Republic of Palau is a small island nation with limited healthcare resources and a lack of onsite subspecialty medical care services such as orthopedic surgery, cardiology, and oncology. Palauans receive sub-specialty healthcare during medical missions from other countries or when they are referred off-island to surrounding countries by the Palau Medical Referral Program. The goal of this study is to identify patterns in costs, locations, and types of cases to elucidate potential areas of improvement to the Palauan healthcare system. **Methods:** This study utilized the 2020–2022 referral data to analyze the frequency of medical conditions that result in off-island referrals and the associated economic burden. Data is presented in a descriptive analysis. **Results**: We found that oncology and cardiology are the two most common types of medical conditions requiring off-island medical referrals and that Palau is spending over 2 million dollars annually for referrals on subspecialty medical care. Cardiology and oncology are the most frequent cases and have the highest costs. The results of this study provide insight into the current state of medical care in Palau. **Discussion/Conclusions**: There is a need for a systematic, timely, and economically feasible approach to subspecialty medical sub-care for Palau, especially cardiology and oncology, to reduce the economic burden for Palau. This approach should be linked with appropriate prevention, risk reduction, and early intervention efforts for these non-communicable diseases.

## 1. Introduction

In the United States, a patient presenting with a fractured wrist typically spends 1 to 3 h in the emergency room (ER) for evaluation, X-rays, and treatment. From the initial ER visit to surgery, the entire definitive process averages 1 to 2 weeks. In contrast, in Palau, a similar patient will spend 1 to 3 h in the ER for initial care; however, the timeline to definitive care extends far longer. The orthopedic consult—often attended by a general surgeon—may take two weeks. If subspecialty surgery is required, a medical referral must be approved by the National Referral Committee (NRC) [1,2]. The timeline for such a process may require 1 to 2 months. As illustrated, this disparity underscores the significant challenge faced by Palau’s healthcare system and the patients relying so heavily upon it.

For island nations like Palau, access to subspecialty healthcare proves to be a persistent challenge. Situated in Micronesia, Palau hosts a population of approximately 18,000 individuals [3]. A limited healthcare infrastructure remains in Palau despite global advancements in healthcare. Lacking local access to specialty care in cardiology, oncology, and orthopedics, Palau is a nation designated as a medically underserved area [2,4]. To address the gaps in locally available healthcare, Palau depends on visiting medical missions and off-island referrals. The Medical Referral Program (MRP), a branch under Palau’s Ministry of Health (MOH) and Human Services, manages off-island referrals made to neighboring countries, including Guam, the Philippines, Taiwan, and the United States (e.g., Hawai’i) on occasion [5]. Representing a critical component of healthcare in Palau, the MRP faces challenges in providing care for the local people.

Overseas medical referrals are common in Pacific countries (Palau, Federal States of Micronesia, American Samoa…) due to a lack of resources and equipment, which are utilized to fill in gaps for specialized care [6]. In 2017, there was a total of 2639 overseas medical referrals in 15 Pacific Island countries with a total combined population of 2,746,950 [6]. Palau was included in this study and contributed 320 cases with a population of 21,729. There is a high incidence of orthopedic cases requiring specialized care, indicating a need for local treatment [7]. This translated to a rate of 14.73 overseas referrals per 1000, which was the third highest rate behind Niue and Nauru, which had rates of 43.75 and 19.05, respectively. [6]. In terms of cost, it was estimated that Palau spent $2,693,227 in 2017 on medical referrals, an increase from $1,935,017 in 2013. [6] Data regarding specialties for Palau were not reported on overseas referrals, but it was noted that on visiting medical teams, cardiology and orthopedics were the most utilized specialized care [6].

Despite these challenges faced by patients, healthcare providers, and the government of Palau, there is limited current research and infrastructure in analyzing the trends, costs, and implications of referrals on the healthcare system of Palau. This study quantifies MRP referral data to reveal the economic and logistical burdens of the referral system. By doing so, this study aims to provide clear and current insights for consideration in policy decisions regarding resource allocation and healthcare reform in Palau. Additionally, the study identifies highly referred specialties that may be prioritized in efforts to recruit specialists or plan medical missions, highlighting opportunities to minimize costs and improve patient care.

## 2. Materials and Methods

The Palau Ministry of Health tracked medical referral claims from 2020 to 2022 and provided us with this data for this study. These claims, received from the Palau Medical Referral Program, were de-identified and contained information relevant to the study, such as diagnosis costs and referral sites before referral.

Prior to analysis, diagnostic codes were processed to stratify referrals into specific specialties. The specialties we considered were cardiology, COVID-19, dental, dermatology, endocrinology, ENT, gastroenterology, general surgery, hematology, hepatology, immunology, infectious disease, nephrology, neurology, OB/GYN, oncology, ophthalmology, orthopedic surgery, plastic surgery, proctology, pulmonology, rheumatology, urology, and vascular. Some referral claims had missing diagnostic codes, multiple specialties listed, or a specific diagnosis provided. We categorized claims without a code under “unspecified”. For claims with multiple codes, we assigned the first code listed unless oncology was present, in which case we prioritized oncology as our area of interest. For the claims that listed a specific diagnosis, we assigned the claim to one of the specialties. For example, we grouped “soft tissue disorder” and “muscle injury” under orthopedics and “prostate” under urology.

After data processing, we conducted a descriptive analysis using Microsoft Excel. The analysis focused on identifying the following statistics: the number of referrals per site and specialty and the total and average costs per site and specialty over a three-year period (2020–2022).

## 3. Results

### 3.1. Types of Cases and Counts of Off-Island Referrals

**Figure 1:**
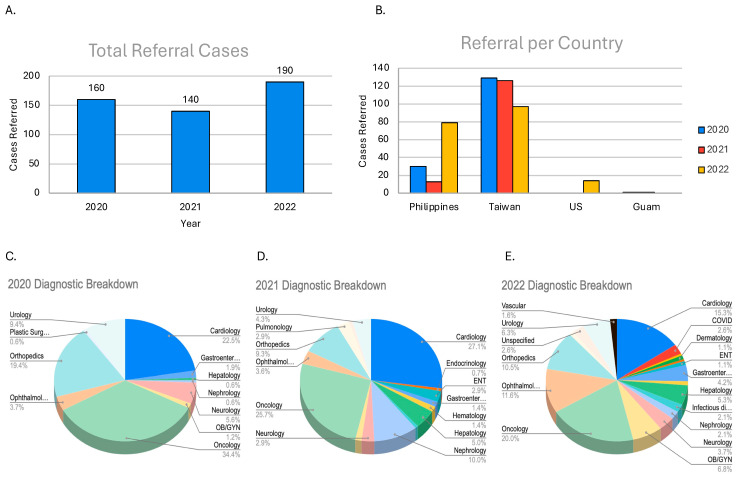
This figure shows the number of cases referred each year from 2020 to 2022 (**A**) and to which site and their frequency (**B**). In addition to the counts of the cases per year, it shows the breakdown of the proportion of each referral category for the three-year span (**C**–**E**).

**Figure 2:**
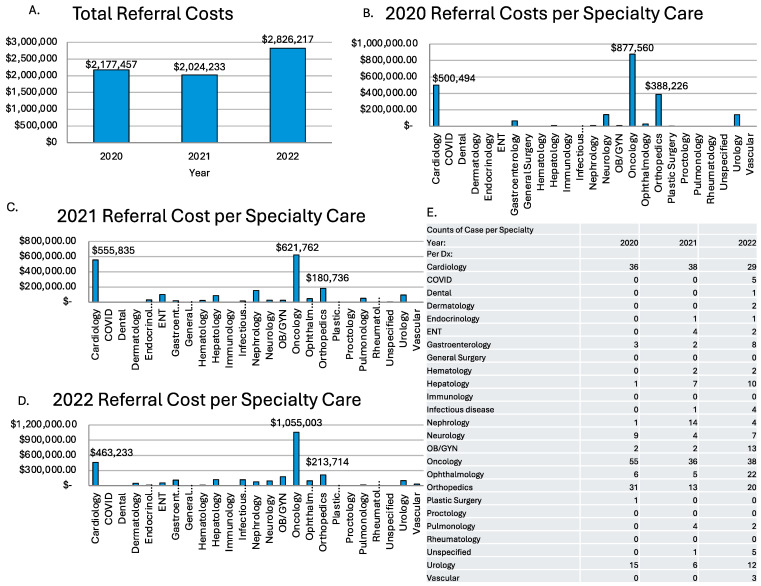
This figure shows the cost of off-island referrals from 2020 to 2022 (**A**) and how much each referral category cost in each year (**B**–**D**). It also shows a table that shows a count of cases for each category in the three-year span (**E**).

During the three-year period (2020–2022), a total of 490 off-island referrals occurred after some cases were excluded from the database. Of these 490 cases, 2022 had the most cases that year, with 190, followed by 2020, with 160, and 2021, with 140 (Figure 1A). There were variations in the types of cases referred across the three years. However, there was a common trend, with oncology and cardiology being the top two specialties’ reasons for referral. Oncology was the most common reason for referral in 2020 and 2022, with 34.4% and 20% of the cases being referred, respectively (Figure 1C,E). This corresponded to 55 oncology cases in 2020 and 36 in 2022 (Figure 2E). Cardiology was another major specialty referred to. In 2021, it was the most common reason for referral (27.1%), followed by oncology (25.7%) (Figure 1D). In 2021, cardiology accounted for 38 cases, while oncology had 36 cases (Figure 2E). In the other years, cardiology was the second most common in 2020 and 2022. It is also worth noting that orthopedic cases were consistently referred off-island for specialty care.

When referred to off-island facilities, Palauans primarily seek specialty care in the Philippines and Taiwan. Taiwan was the most utilized site for specialty care in the 3-year period, while the Philippines was the second most used site (Figure 1B). In 2022, there was an increase in cases being referred to the Philippines, almost making it the most common site that year. There were some cases being referred to the U.S. and Guam, but those cases made up a small percentage of total cases.

### 3.2. Costs of Off-Island Referrals

In those three years of referrals, it cost the Palauan government and people a total of $7,027,096.67. The costliest year was 2022 due to the increased number of cases being referred; in 2022, it was $2,826,216.66, followed by 2020, with $2,177,457.18, and 2021, with $2,024,232.83 (Figure 2A). The total costs for each specialty category were calculated. It was found that since oncology was the most common reason for referral, it was also the costliest referred specialty care. The sum totals for oncology by year were $877,560.39 in 2020, $621,761.79 in 2021, and $1,055,002.59, the costliest, in 2022 (Figure 2B–D). This was notable because 2022 was the costliest year despite having 17 fewer cases than 2020 and only two more cases than 2021. This resulted in 2022 having the highest average cost for oncology. As mentioned previously, cardiology was another common reason for referral and was also among the costliest specialties. It was the second most costly reason for referral in all three years. The total costs per year were $500,493.76 in 2020, $555,834.56 in 2021, and $463,233.29 in 2022 (Figure 2B–D). It is also worth mentioning that orthopedics also made up a good portion of the yearly cost for referrals due to the consistency of cases being referred. Overall, oncology accounted for a large portion of annual referral expenditures due to the high number of cases and the high average cost per case.

## 4. Discussion

Our study found that the most common and costly off-island referrals from 2020 to 2022 included cardiology and oncology patients. Ideally, building upon the local infrastructure in Palau would help establish a more self-sustainable healthcare system and reduce the need for off-island referrals. However, islands of the Pacific face numerous barriers, including a small, dispersed population that may not sustain a practice for specialists; low reimbursement rates for healthcare workers; and high costs for equipment, facilities, and their maintenance [8]. Currently, Palau lacks the resources for cardiology and oncology specialists, diagnostics, and treatment facilities. Possible ways to reduce the need for off-island referrals include increasing the number of visiting physicians via medical missions or invest in training local healthcare workers to provide more specialized care.

Another area of great interest is the prevention and screening of cardiovascular disease and cancer. Palau is at a higher risk for cardiovascular diseases and cancers due to increased rates of obesity, hypertension, diabetes, and betel nut chewing. Rising rates of obesity, hypertension, and diabetes may be attributed to the shift from an indigenous to a Western diet [9]. The common types of cancers seen in Palau are pulmonary, liver, prostate, oral and pharynx, uterus, colorectal, breast, cervix, thyroid, and stomach [3]. The 2023 Palau Hybrid Survey found that approximately three out of four adults were overweight or obese [10]. Almost half of adults were classified as having hypertension, with over half of these adults being undiagnosed. Of those previously diagnosed and on medication, more than half remained uncontrolled. The study also found a high prevalence of diabetes, of which the majority of patients were also underdiagnosed and uncontrolled. This is important, as obesity, hypertension, and diabetes are major contributors to cardiovascular disease [11]. Betel nut chewing is a significant health concern in Palau due to its link to oral cancer. Almost half of Palauan adults reported chewing betel nut, with the majority doing so daily and adding tobacco to their chew [10,12]. Implementing more regular monitoring can improve the management of these chronic conditions. For example, this can be achieved through recruiting more healthcare providers, developing telemedicine sites, expanding local health worker training programs, or increasing outreach days to outlying islands to enhance accessibility [13,14]. The training and retention of local healthcare providers have long been challenges in providing specialized care in Palau [14,15]. Education on healthy lifestyles and the implications of these chronic diseases may also help with prevention efforts. Prevention of chronic disease starts at the local level with education, housing, nutrition, and other social factors [16].

Screening and early cancer detection are among the many prevention strategies that can reduce cancer-related deaths by up to 50% [17,18]. However, screening efforts in Palau have faced challenges in the past. Despite the implementation of the Breast and Cervical Cancer Early Detection Program (BCCEDP), only 64.5% of adult women were up to date on their Pap smears, and 36.6% of adult women were up to date on their mammograms [8]. Challenges with screening include a shortage of resources and accessibility, lack of awareness of screening and symptoms to look out for, and low comfortability with screening providers [19,20]. More culturally sensitive screening processes must be developed to improve early detection and treatment of highly detectable cancers, such as uterine, breast, prostate, and colorectal cancers.

In addition to identifying the most common reasons for referral, we found that Taiwan and the Philippines were the most frequently used referral sites. We suggest possibly exploring partnerships with these neighboring countries to find ways to reduce costs.

Future directions include assessing Palau’s current medical capacity and ongoing efforts to reduce medical referrals and improve local care. Exploring policies of other Pacific Island countries to identify partnerships and successful programs that could be emulated. In addition, continual monitoring of referral cases is needed to track trends for specialty care needs. A key limitation of our study was reliance on a single dataset with limited details on logistics, cost breakdowns, patient experiences, and short- and long-term patient outcomes. With more detail, the effectiveness of the MRP and a more specific cost analysis could be determined.

## 5. Conclusions

Palau utilizes the off-island referral system at a high capacity for many of their specialty care cases. At the conclusion of the study, we found that oncology and cardiology were the top two referral categories from 2020 to 2022 in terms of cost and number of referrals. The majority of cases are sent to Taiwan and the Philippines for care, with fewer cases sent to the U.S. or Guam. Over the past three years, off-island referrals have cost over $7 million. Compared to 2017, fewer cases were referred, but costs remained relatively the same, which may be attributed to the rising cost of treatment. This study could serve as a guide for adopting oncologic or cardiovascular interventions to reduce the economic burden of referrals. Enhancing medical infrastructure to support oncological treatment and diagnosis could be a cost-effective long-term solution for Palau. This could be achieved by acquiring new equipment and training current medical staff in advanced procedures.

## Data Availability

The original contributions presented in this study are included in the article. Further inquires can be directed to the corresponding author.
